# Perceptions of Long COVID Patients Regarding Health Assistance: Insights from a Qualitative Study in Spain

**DOI:** 10.3390/nursrep14040243

**Published:** 2024-11-04

**Authors:** Maria Leopolda Moratalla-Cebrian, Irene Marcilla-Toribio, Carlos Berlanga-Macias, Ana Perez-Moreno, Maria Garcia-Martinez, Maria Martinez-Andres

**Affiliations:** 1 Health and Social Research Centre, Universidad de Castilla-La Mancha, 16071 Cuenca, Spain; leopolda.moratalla@uclm.es (M.L.M.-C.); ana.perez22@alu.uclm.es (A.P.-M.); maria.martinezandres@uclm.es (M.M.-A.); 2 Faculty of Nursing, Universidad de Castilla-La Mancha, 02071 Albacete, Spain; 3 Research Group Health, Gender, and Social Determinants, Instituto de Investigación Sanitaria de Castilla-La Mancha (IDISCAM), 16071 Cuenca, Spain; 4 Gerencia de Urgencias, Emergencias y Transporte Sanitario (GUETS), 45071 Castilla-La Mancha, Spain; 5 Simulation, Life Support, and Intensive Care Research Unit (SICRUS), The Health Research Institute of Santiago de Compostela, 15706 Santiago de Compostela, Spain; maria.garcia.martinez0@rai.usc.es

**Keywords:** long COVID, COVID-19, healthcare, quality of life, symptomatology, public health, qualitative research, nursing

## Abstract

Objective: This study investigates the perceptions of Long COVID patients in Spain regarding the healthcare they receive to identify demands and areas for improvement. Methods: Using a qualitative descriptive phenomenological approach, the study included 27 participants selected through non-probabilistic convenience sampling. Data were collected via online semi-structured interviews and analyzed using thematic analysis. Results: The findings reveal three key themes: (i) health status and challenges in healthcare during the initial COVID-19 infection; (ii) perceptions about healthcare as Long COVID patients; and (iii) demand for and aspects of improving quality of healthcare. The participants, predominantly women (66.67%) with a median age of 51 years, experienced symptoms that they generally perceived as severe, although only 14.81% required hospitalization. The participants reported initial self-management of symptoms at home, which was influenced by familial responsibilities and hospital overcrowding, and the persistence of a wide range of Long COVID symptoms that significantly impacted their daily lives. Satisfaction with healthcare services varied, with frustrations over systemic inefficiencies and long waiting times. Conclusions: The study highlights the need for timely access to medical care, comprehensive and empathetic healthcare services, and specialized Long COVID units. The results emphasize the importance of patient-centered approaches and multidisciplinary care to address the complex nature of Long COVID effectively. These findings provide crucial insights for improving healthcare protocols and systems to better support Long COVID patients. This study was prospectively registered with the Ethics Committee for Research on Medicines of the Albacete Integrated Health Care Management System (registry) on 22 February 2022 with registration number 2022/001.

## 1. Introduction

The COVID-19 pandemic has had a significant global impact, disrupting various aspects of life and causing widespread challenges to public health and economies worldwide [1]. In Spain, during the initial five waves, approximately 5 million cases were reported, leading to over 431,891 hospitalizations, 41,138 ICU admissions, and 87,080 deaths [2]. Despite previous warnings from pandemics such as the 2009 influenza and SARS-CoV-1 epidemic, Spain, like many other countries, was inadequately prepared in January 2020. The absence of strategic reserves, coupled with weak information systems and insufficient diagnostic resources, has resulted in one of the highest global increases in mortality and a notable decline in life expectancy [2]. This scenario has prompted a reevaluation of crisis preparedness, highlighting that high rankings in the Global Health Security Index do not equate to effective pandemic management [2].

In addition to the immediate health crisis and economic disruption it created, the pandemic has resulted in the long-term health complication termed Long COVID, placing a substantial burden on health services. Long COVID is defined by the persistence of symptoms for months following the acute phase of the disease. According to the World Health Organization (WHO), Long COVID is characterized by signs, symptoms, or abnormal clinical parameters that persist for three months after the onset of COVID-19 and continue for at least two months without an alternative diagnosis [3]. It is estimated that Long COVID affects between 10% and 35% of COVID-19 survivors [4].

The symptomatology of Long COVID is varied and can affect multiple organ systems, resulting in a heterogeneous clinical presentation. Common symptoms include fatigue, cognitive difficulties (often termed “brain fog”), shortness of breath, headache, joint and muscle pain, and emotional distress, such as anxiety and depression [5,6,7,8,9]. Over 200 possible symptoms have been identified, impacting the general, respiratory, cardiac, neurological, psychological, otorhinolaryngological, ophthalmological, dermatological, and digestive systems [10,11,12]. The risk factors for developing Long COVID include being female, being old, and having a severe initial infection [11,12,13,14,15].

Currently, there is no standardized approach for diagnosing or managing Long COVID, and no effective and approved cure is known [5,16]. In addition to the aforementioned high economic impact of Long COVID—due to factors such as increased healthcare spending and social security costs for sick leave—the quality of life of those who suffer from this illness can be severely affected, and it can be tremendously disabling in some cases [10,17]. In this context, to date, most studies have focused on the symptomatology and potential treatments for Long COVID, but few have considered Long COVID from the perspective of the patient and the healthcare received. Nevertheless, initial evidence suggests that patients have a poor perception of the care they receive, including poor recognition of their condition [18]. In fact, the lack of recognition and support systems has driven many patients to seek alternative support, which can sometimes have counterproductive effects [19]. This underscores the need for improved, patient-centered care, including psychological support and self-management strategies, to address the complex and multifaceted nature of Long COVID [20,21].

This article aims to complement previous research and enhance existing knowledge about healthcare system functioning in Spain in treating Long COVID patients through a qualitative approach based on patients’ experiences. Specifically, the objective is to describe the perceptions of Long COVID patients regarding the healthcare they have received since their initial infection. This paper aims to answer the following research questions:

▪ How do Long COVID patients perceive the healthcare services they have received in Spain?
▪ What are the main challenges and areas for improvement in the current healthcare system according to Long COVID patients?
▪ How do these perceptions affect their overall satisfaction with the healthcare system?

## 2. Materials and Methods

### 2.1. Design, Setting, and Population

This is a qualitative descriptive phenomenological study conducted in Spain. The study is part of a larger cross-sectional and observational research project that investigates the impact of Long COVID symptomatology on patients [22]. The descriptive phenomenological approach was the most appropriate for understanding the experiences of our participants with Long COVID and guided the study’s design, data collection (semi-structured interviews), and data analysis [23,24].

The research was conducted in a virtual setting, using online questionnaires in the cross-sectional part and video calls on Microsoft Teams version 6 (Microsoft Company, Washington, USA) due to ongoing restrictions during the pandemic, facilitating the participation of this geographically dispersed population. The population targeted for this study included individuals who had experienced Long COVID symptoms for three or more months. Non-probabilistic convenience sampling was used after contacting the participants of the cross-sectional and observational research (145 people, 113 women and 32 men) by e-mail.

The inclusion criteria were adults aged 18 years or older who had been diagnosed with COVID-19 or had experienced symptoms compatible with COVID-19 for at least three months. The participants also needed to be fluent in Spanish (reading, writing, and speaking). The exclusion criteria included individuals who were unable to participate due to technical limitations or language barriers. Finally, 27 people participated, a sample size defined by the saturation of information criteria [25]. Of these, 18 were women and 9 were men, belonging to different regions of Spain.

### 2.2. Data Collection

The semi-structured interviews were conducted and recorded via videoconference using Microsoft Teams Classic by the same interviewer (ML. M-C), a researcher with experience in qualitative methodology and communication skills, who established a relationship of trust that facilitated free dialogue and communication. The possibility of holding interviews on several different dates and times of the day was offered to create a pleasant atmosphere that would facilitate the verbalization of their experiences [25,26]. The semi-interviews lasted between 27 and 68 min, with an average duration of 42 min, and were conducted between October and December 2022.

As a part of the data collection process, each interview began by asking participants to rate their current health using a visual analogue scale (VAS) from 0 to 100, where 0 represented “the worst imaginable health state” and 100 represented “perfect health”. This helped frame the discussion and provided context for the severity of their symptoms and overall health perceptions at the time of the interview. The interviews were conducted on the basis of a preliminary script with the most significant aspects defined in the previous documentation work. Furthermore, two pilot interviews were conducted to assess their suitability [26]. The script focuses on four main sections: (i) experiences with Long COVID, (ii) working settings, (iii) healthcare services, and (iv) social settings and support. In the present study, our analysis has focused on healthcare services. Table 1 shows the interview guide.

The interview period concluded following the saturation of information criteria. The interviews were transcribed and read to extract the most relevant ideas. Thus, a summary of these ideas was undertaken and shared with the participants so that they could assess whether this summary responded to the considerations that these people proffered during data collection. None of the participants needed any clarifications nor mentioned that they did not feel represented by the ideas disclosed.

### 2.3. Data Analysis

The qualitative data were analyzed using Braun and Clarke’s method of thematic analysis [27], which is an inductive approach designed to capture and reflect participants’ experiences without imposing predetermined theoretical frameworks. ATLAS.ti software, version 24.1.1 (ATLAS.ti Scientific Software Development GmbH, Berlin, Germany) was used to facilitate the coding and organization of the data. Three experienced researchers (M.L.M-C, I.M-T, and M.M-A) independently coded the data, identifying themes from the participants’ narratives. The coding process was conducted in three stages: generating initial codes from the raw data, grouping these codes into broader categories, and identifying overarching themes on the basis of these categories (Table 2). This process ensured that the analysis remained grounded in the data and aligned with the descriptive phenomenological approach.

### 2.4. Ethical Aspects

The study protocol was registered and approved with number 2022/001 by the Ethics Committee for Research on Medicines of the Albacete Integrated Health Care Management System. All research procedures used in this study were established in accordance with the Declaration of Helsinki. All the participants gave their consent to participate in the study after they were duly informed about its purposes and procedures. The participants who were interested had to select whether they wanted to participate in the cross-sectional part, the qualitative part, or both when filling out the online consent forms. After completing the cross-sectional study, the research team contacted these individuals via e-mail. At the beginning of the interviews, the participants were reminded that the interviews would be recorded and that they would be assigned an identification code to anonymize their data. The participants were also reminded that they could revoke their consent at any point during the research process.

### 2.5. Quality and Rigor

This study followed the COREQ (Consolidated Criteria for Reporting Qualitative Research) reporting criteria [28]. Several strategies have enabled us to ensure a meticulous analytical approach. The interviews were previously piloted with people who fulfilled the inclusion criteria and were conducted by the same experienced researcher (M-L. M-C). In addition, the research teams reviewed and validated the interviews script to eliminate interpretation bias. The scripts had open-ended questions to facilitate the free speech of the participants. Furthermore, the forwarding of a summary of the information obtained from the participants permitted them to review the content to validate or reject the main conclusion. Finally, analysis and coding with three different researchers at three degrees of depth allowed for subsequent discussion of possible discrepancies and resolution via consensus [29].

## 3. Results

Table 3 summarizes the key sociodemographic characteristics of the 27 participants. The majority were women (66.67%), with a median age of 51 years. Most participants had completed tertiary education (55.56%), and 59.26% were on sick leave for more than three months due to Long COVID symptoms. The majority of participants were diagnosed with COVID-19 via a PCR test, and while 51.85% considered themselves to have experienced severe symptoms, only 14.81% had required hospitalization. In Table A1, a detailed breakdown of the Long COVID symptoms experienced by the participants can be consulted, specifying their intensity. Among the most frequently reported and intense symptoms were fatigue, muscle and joint pain, and brain fog.

### 3.1. Health Status and Challenges in Healthcare During Initial COVID-19 Infection

#### 3.1.1. Management of Initial Symptoms and Access to the Healthcare System

Although the majority of participants perceived their symptoms as severe, many initially reported experiencing symptoms that they likened to a mild cold or flu at the onset of the infection. This led them to initially manage these initial symptoms at home (P05: “When I started experiencing COVID symptoms, it’s true that I’ve felt worse with any gastroenteritis or severe flu. I felt worse than with COVID; it wasn’t very hard to get through.”; P06: “I knew I had pneumonia because I had bronchitis with early-stage pneumonia about five years ago, so I roughly knew how it felt. But I wasn’t feeling bad enough to go to the hospital, so I stayed at home”). However, some interviews revealed various barriers, whether familial or social-health-related, in receiving the best possible healthcare. Some participants, especially women, indicated that they dismissed the possibility of going to the hospital despite experiencing symptoms perceived as severe because they felt obligated to care for close family members (P04: “Apart from the general discomfort, I had a terrible headache and bone pain. However, the situation at home was difficult; my husband was quite ill and had been in the ICU. At that time, my daughter also got infected, so I had to stay strong.”; P20: “I called, and they told me, ‘You have COVID, stay at home’, and that was it. I stayed home for a few days, and no one called to check on me or see how I was doing. As I was getting worse, I called again. Then, they suggested doing an X-ray to see how I was. They took me for an X-ray and told me there was no bed available and that I had some questionable inflammation. They weren’t sure what it was. I went home because I said I didn’t want to be admitted and worry my son, so I came back home”).

On the other hand, some participants were forced or chose to stay at home because of hospital overcrowding or recommendations from health services or information from the media advising against going to emergency rooms (P10: “I stayed at home because there were no beds available. I was aware of the situation. They told me they would admit me to the ICU, but if anyone was aware of the hospital occupancy, it was me. I had been working in X and Y hospitals [blinded to ensure anonymity], and I knew there were no beds, so I went back home”; P11: “After 3 days, I developed symptoms compatible with COVID. At that moment, the healthcare providers completely disregarded me because I tested negative for COVID, and given the situation in the hospitals, if you weren’t COVID positive, they would ignore you entirely”; P01: “In the end, I developed bilateral pneumonia, and it was very bad. I live alone, and the advice I received, both when I went to the hospital where it was diagnosed and from my colleagues in primary [care], was that it was better to stay at home given the chaos in the hospitals at that time. So, that’s what I did”; P21: “When I arrived at the hospital, they did a PCR test [...] it came back positive. Well, at that time, it was September 28, 2020, and the situation was quite turbulent. The doctor who attended to me said, ‘I’m not going to admit you [to hospital] because we are extremely overwhelmed. It’s better for you to recover at home. We will monitor your situation and assess it as we go’, which is what they were doing back then”).

#### 3.1.2. Experience in Accessing Diagnostic Tests

The testimony of a significant number of interviewees highlighted the unreliability of the initial detection tests and how many of them had to undergo numerous tests before testing positive (P02: “The first one to test positive was my husband. My daughters and I took a PCR test that same day and tested negative. Three or four days later, I started having a severe sore throat and cold-like symptoms. The doctor came to see me, did another PCR test, and it came back negative again. Then my husband, about a week later, began to have difficulty breathing and went to the hospital. That same day, my daughters and I were given another PCR test, and I had already had a severe headache since the day before, Sunday (this was Monday), and I started to develop a fever. I already suspected that I had contracted it, and indeed, the PCR test came back positive”; P08: “Initially, they did us a PCR test, which came back negative for me, but it was one of those early tests that often failed. Later, they did more tests, and I started testing positive”).

In this regard, many participants emphasized that their diagnosis was based on the clinical picture, which led the physicians to prescribe additional diagnostic tests or even to diagnose COVID-19 regardless of the test results (P03: “They did a blood test and a PCR, and both came back negative, but since the doctor at the time wasn’t comfortable with the pneumonia I had, she said they would repeat the tests. When they repeated them, it turned out that I had COVID”; P17: “I was in the hospital for two weeks, and they couldn’t get a positive test result […] “Then, there came a point when, since I didn’t have a positive test result, they were going to discharge me. As a precaution, on the day they were going to discharge me, they did a CT scan. The pattern of lung damage caused by pneumonia must be very specific to COVID compared with other pneumonias, and everyone got alarmed. They reversed the decision, isolated me even more than before, and started over. They officially diagnosed me as COVID positive even though it hadn’t shown up in the PCR test”).

Another issue highlighted by the interviewees was the widespread use of antigen tests within the home setting due to difficulties in accessing PCR tests. The significance of this fact is twofold: on the one hand, the tests have lower reliability compared to PCR tests; on the other hand, the results of these tests were not officially recognized (P19: “Being a healthcare worker, I was supposed to get a PCR test, but they didn’t do it. I tested positive with an antigen test. So, I went to my primary care doctor. She monitored me over the phone, not in person. I requested a PCR test, but they refused to do it. Then, since I was no longer testing positive in the third week, they discharged me, even though I was feeling very unwell […] During this time, I had already been to the emergency room three or four times. After 5 weeks, they did a PCR test, and it came back positive. That was the first PCR test they did for me because until then, they hadn’t done any”).

### 3.2. Perceptions About Healthcare as Long COVID Patients

#### 3.2.1. Evolution of Long COVID Symptoms and Initial Medical/Domiciliary Care

The evolution of Long COVID symptoms in both men and women reveals a persistent and debilitating impact on their daily lives. Initially, patients experienced severe fatigue, muscle and joint pain, and headaches, which continued to affect them long after the acute phase of infection (P07: “Currently, the most intense symptoms are fatigue and muscle pains. I also have a sleep disorder and take medication nightly to sleep”). Cognitive issues, such as memory loss and concentration difficulties, were also prevalent (P01: “On a cognitive level, it’s horrible. It’s like suddenly aging 20 or 30 years because you forget things and words don’t come out”). These neurological issues often required patients to find coping mechanisms, such as using notes and reminders to manage daily tasks (P07: “My house is full of post-it notes, and I have a whiteboard where I write things down”).

As time passed, many patients continued to suffer from cardiovascular and respiratory symptoms, such as palpitations and shortness of breath (P08: “I started with extreme fatigue and ventricular extrasystoles”). Dermatological issues, including hair loss and skin rashes, were also reported (P01: “I now have psoriasis and psoriatic arthritis”). The psychological toll was significant, as many patients faced anxiety, depression, and cognitive difficulties, impacting their overall quality of life (P10: “I often get stuck and can’t remember things, which has made me lose my hobbies”). Some digestive issues, such as intermittent diarrhea and stomach pain, persisted. (P10: “I have to be very careful with my diet because I have intermittent diarrhea”). Overall, Long COVID patients continued to endure a broad spectrum of symptoms that severely affected their physical, neurological, and emotional well-being.

Concerning the type of symptoms and their influence on healthcare assistance, long COVID patients perceive that the care they receive is heavily influenced by whether their symptoms are observable or objectively measurable. They feel that when their symptoms are considered subjective, a barrier is established, and the healthcare system tends to ignore or undervalue their needs (P:13: “I don’t know if it’s an advantage or what, but presenting with a visible symptom makes a difference. I couldn’t speak properly, and the attention I received was completely different. My experience with the post-COVID unit and all professionals has been excellent, unlike others with less visible symptoms. Fatigue is common among us, but it isn’t seen, whereas my voice is heard, so my experience has been good”). Among those with symptoms considered non-objective, feelings of frustration and rejection predominated (P05: “I’ve ended up feeling like, because it isn’t visible, it seems like you’re fine”; P09: A third participant expressed, “It’s difficult because you appear to be more or less okay. So, for them, it’s hard to explain… I can’t keep complaining all the time that I’m tired. It’s hard to explain”; P16: “When you go to the doctors and everything appears perfect, but you feel terrible, you think, ‘I’m not making this up, it hurts’. Nothing shows up, but I feel awful, and they can’t find anything. It’s very frustrating”; P11: “Those of us with headaches feel somewhat ignored by the system. The problem is that it’s very, very subjective, and there’s no way to objectify what we have. For those of us with headaches, it’s a problem”).

Finally, to gauge the perceived health status of the patients, they were asked to self-report their health status on a scale from 0 to 100 (EVA). The results varied significantly, reflecting a broad range of experiences among the participants. Female participants reported scores ranging from as low as 3 (P07, P20) to as high as 70 (P03, P05, P13, P19), with many others reporting intermediate values such as 60 (P01, P04, P09) and 50 (P10, P16, P24). This variation suggests that while some female participants feel relatively better, others continue to experience significant health challenges. Male participants also showed a wide range of scores. P11 reported the highest score of 90–95, indicating a better perceived health status, whereas P12 and P22 reported much lower scores of 25 and 15, respectively, suggesting ongoing health issues. Other male participants, such as P08 and P14, reported intermediate values (65–70 and 50–60, respectively). The self-reported scores highlight the significant individual variation in perceived health status among Long COVID patients, with both genders showing a range of experiences from severe health impacts to relatively better recovery or management of symptoms.

#### 3.2.2. Satisfaction with Healthcare

Patients had mixed experiences with primary and specialized healthcare. While some expressed satisfaction with their personal interactions with the physician (P07: “I was very tired with a lot of pain, I didn’t have a fever. I’m from a small village, and the truth is that when I caught it, I was the only one infected, and my primary care doctor called me a lot. I have a very good relationship with him”), many were frustrated with the system’s overall inefficiencies and delays, criticizing aspects such as the impossibility of more personal treatment (P20: “From the beginning, primary care was telephone-based and remains so today. Imagine phone care when you’re saying you’re suffocating, have a terrible headache every day, and your bones hurt. They tell you to take paracetamol and call it back in ten days. It has been very negative and a torment, not feeling cared for, consoled, or improved”; P17: “…because the healthcare staff didn’t know how to handle us and wouldn’t come in. I spoke to doctors over the room phone. It was very hard, isolating… the uncertainty, feeling like I could die and having no support except by phone”) or the difficulty of scheduling appointments within an acceptable timeframe (P19: “It has happened to me with all specialists, getting dates for four months, eight months, nine months, five months later”).

In this regard, the psychological impact of Long COVID and the perceived lack of support from healthcare providers were significant issues for many patients (P18: “The neurologist initially told me she couldn’t help. Imagine the disappointment when a doctor tells you this. I understand everything is new, but when you’re that ill and hear that, it’s very frustrating”). Overall, the participants, despite the difficulty of their situation, show understanding of the challenges that medical teams faced in providing solutions due to the novelty of the disease. However, in some cases, they reported experiencing situations in which healthcare professionals rejected the diagnosis of Long COVID (P21: “I’ve encountered doctors with significant disrespect, leaving consultations crying. They would say things like, ‘This is like fibromyalgia,’ implying it was all in my head. When you feel this bad and hear that, it’s devastating”; P12: “… you always encounter people who deny the existence of Long COVID, whether they are doctors or ordinary people”). Some patients turned to private healthcare due to the limitations of the public health system in providing responses to patients’ needs (P05: “I initially went to primary care, but when no solution was provided, I turned to private specialists through my private insurance for thorough examinations”; P07: “I go to a private neurologist to address sleep issues because the public health system discharged me, saying that my mental fog was not their concern”).

When evaluating the healthcare received by Long COVID patients, participants were asked whether they had been informed or treated by nurses as part of the care for this condition. The responses indicated that nursing services did not play a significant role in the healthcare response to Long COVID. Most participants reported that their interactions were primarily with physicians, with minimal involvement from nursing staff, who were often limited to tasks such as drawing blood for tests or administering vaccines (P03: “So far, only doctors. Nothing else, apart from blood tests”; P05: “None, none, none. Beyond a couple of times I had to go to the ER, and they did the triage”. This sentiment was echoed by others, indicating that their experiences with nursing staff were largely incidental and not integrated into their ongoing Long COVID care (P04, P06, P09, P10, P07).

With respect to satisfaction with healthcare services, there was a broad consensus among participants who were referred to specialized Long COVID units, who positively evaluated them (P06: “When the healthcare system organized specialized Long COVID consultations, I was referred to my reference hospital. I switched to a unit that was functioning well, where an internist was very involved and supportive”). However, other participants described the barriers that they encountered when trying to access these units, either due to selection criteria (P20: “I tried to get into a Long COVID unit but couldn’t. They only admitted those who had been hospitalized. I even wrote to the ombudsman and was finally directed to another city for care”; P01: “I asked if there was a possibility of getting into the Long COVID units that were created for follow-up, but they only admitted those who had been hospitalized, they didn’t admit anyone else”) or the lack of specialized units in some regions (P26: “Here there are no [specialized units], the most specific they could offer me was internal medicine.”). Another aspect highlighted by the participants was the lack of information among primary care doctors about Long COVID units and their functioning. This, naturally, complicates the proper referral of patients, although this gap has been to some extent mitigated by the actions of Long COVID patient associations. (P10: “I had to ask my primary care doctor, as they told me in the association that there was a [specialized] unit. He said, ‘Wait, I need to find out.’ He didn’t even know how to refer me. The man just didn’t know”.)

### 3.3. Aspects of Improving Quality Healthcare

#### 3.3.1. Proposals for Improving the Quality of Resources and Treatments

The demands of Long COVID patients regarding improvements in resources and services for better care of this disease can be particularly articulated in two ways: the possibility of having medical appointments with specialists within shorter timeframes, and the creation of specialized units that facilitate coordinated and efficient work among the different healthcare services.

Patients with Long COVID strongly emphasize the need for significantly shorter waiting times for medical appointments. They argue that long delays in scheduling tests and referrals to specialists exacerbate their condition, leading to further deterioration of their health (P03: “What I would add is that it shouldn’t take so long. It takes a long time to do the tests, it takes a long time to refer to specialists, so of course, if you are feeling worse each time and they keep delaying your appointment, you will end up getting worse”; P10: “The psychologist will see me in February, and then it’s the only time he has seen me, the poor guy apologizing, I almost had to console him because he said it was impossible to get here after 2 years”; P19: “That’s very tough […] But it has happened with all the specialists who gave me appointments for four months, 8 months, 9 months, 5 months later”). Due to these long waiting times, as previously mentioned, some patients have resorted to private healthcare to receive timely treatment (P19: “I had to go private because with public healthcare, they gave me an appointment for 9 months later, which is not feasible”).

Some participants suggested that healthcare should be more accessible in each health region, emphasizing the need for regional references for patients due to long waiting lists (P27: “I think what should be done is to bring it closer to each health region. I’m not saying in every regional hospital, but there should be references for patients because there are long waiting lists and many people are left out. For example, I started follow-up a year after having the symptoms, but there are many people who started much later”).

Consequently, a priority for patients would be the creation of more Long COVID units closer to their health areas. These units should be multidisciplinary, coordinated, and include specialists trained in such fundamental aspects as diagnosis, symptoms, and the course of the disease. In addition to the multidisciplinary nature of the care, there is a call for the generalization of these units to ensure that patients do not have to go from one place to another (P26: “It should already exist in any reference hospital of the public health system, that a department is created to address this, and that in a multidisciplinary way, it connects the main specialists who ultimately see us, instead of us running around”).

The participants’ statements contain a critique of the current level of training of healthcare staff regarding Long COVID, even in specialized units (P03: “I noticed that they are not updated or don’t know, or don’t want to know, I don’t know […] You end up wondering why they sent you to the Long COVID unit if the specialist has no idea”; P05: “There should be more answers and healthcare professionals who are sensitized. They need to update their knowledge because I’ve clearly noticed the difference between those who have read about it and those who knew nothing”; P19: “By creating a real Long COVID unit and truly training all specialists, because the specialists don’t know what Long COVID is”).

Understanding the symptoms is a fundamental part of the care that these units should provide, treating Long COVID as a serious illness with multidisciplinary teams (P05: “First of all, I think it’s important to know what the symptoms are”; P20: “So first, treat it as the disease it is, because it is a disease, and try with a multidisciplinary team to alleviate the possible symptoms and have serious research because there are many of us”). The treatment should also include clear communication and explanations, which are crucial for patient understanding and mental well-being (P25: “They should give you guidelines, explain what it is, first explain and know what’s happening to you. I still don’t know what’s happening to me, more than from Long COVID groups, sharing information, listening to them, watching lectures, I see everything that exists because I need to know what’s happening to me”: P05: “Having an explanation helps you understand, and if you understand what’s happening to you, you stop asking questions and worrying. Knowing it’s not you, knowing these are symptoms that occur and understanding why helps”).

The participants also mentioned the need for integrated teams, including respiratory and physical physiotherapists, to help recover lost functions and a good psychopedagogical team to aid in relearning (P10: “An integral group of professionals, especially respiratory and physical physiotherapy to try to recover all that we have lost and a good psychopedagogical office to relearn because you lose a lot”; P19: “A Long COVID unit should be a unit where all specialists know about Long COVID, like a small COVID hospital, so there would be a neurologist who knows, who is trained, a rheumatologist who also knows, a physiotherapist… what I’ve missed the most is physiotherapy”).

#### 3.3.2. Improvements in the Humanization of Healthcare Teams

Patients with Long COVID report not only physical problems but also associated psychological issues. Thus, the official recognition of the disease represents a critical step, both to in terms of being able to provide effective treatment and the mental relief it brings to the patient (P09: “So first, that the disease is recognized, that they make diagnoses of Long COVID”; P26: “I think the diagnosis alone helps a lot, just telling the person who what they are experiencing is real, that they are not imagining it, that they are not going crazy, that it exists”; P05: “Having an explanation already helps you understand, and if you understand what is happening to you, you stop asking questions and stop worrying”). In the same vein, participants call for the need to humanize care and have a sufficiently empathetic healthcare team that can listen to and understand patients’ situations and provide support. The care should be comprehensive and include non-pharmacological treatments or therapies, such as physiotherapy or psychological consultation (P10: “I think it is very important that the professional in front of you has empathy with you”; P13: “A little empathy, to listen to you, and not to attribute everything to mental problems, depressions”; P25: “I understand that not much is known about the topic yet, and I understand that they cannot give us solutions, but at least listen to us”).

## 4. Discussion

The main objective of this study was to analyze the perceptions of Long COVID patients from Spain regarding healthcare to identify patient demands and potential areas for improvement in the healthcare system. The data analysis highlights three key themes: (i) understanding the progression and healthcare experiences of Long COVID patients since their initial infection, (ii) analyzing their level of satisfaction with healthcare, and (iii) identifying demands and aspects for improving the quality of healthcare on the basis of their experiences.

Most participants reported experiencing mild symptoms initially, similar to a cold or flu, which led them to manage their symptoms at home. This finding aligns with previous studies that have shown that many COVID-19 patients initially treated their symptoms without seeking immediate medical attention [30]. However, familial and social health barriers prevented some, especially women, from seeking hospital care despite severe symptoms. This result aligns with the observation that women, who are often responsible for domestic well-being, tend not to prioritize their health treatment over their family responsibilities and feel selfish if they place their own needs above those of their family. This reality was exacerbated by the pandemic, as the responsibility for care fell primarily on women throughout the two years of the pandemic [31].

Additionally, some participants avoided hospitals because of overcrowding and the recommendation to stay home unless they were critically ill, as previously reported [32]. This mirrors findings from studies conducted during the pandemic, which reported similar patient behavior due to fear of hospital overload and following public health advisories [33]. The experience of Long COVID patients in Spain, characterized by hospital overcrowding and limited access to specialized care, could reflect what has happened in other countries. For example, in regions of the United Arab Emirates [34], China [35] and Iran [36], a sharp reduction in healthcare utilization has been observed, similar to that observed in Spain.

The main pathways behind the reported reductions could be related to the exacerbation of pre-existing barriers, such as changes in both the amount and distribution of resources, legal or discriminatory barriers, and the access to accurate information [37]. Similarly, the introduction of telecare modalities may have led to inequalities in access, probably as a result of the lack of digital literacy and material resources [37]. On the other hand, the fear of contagion and the stigma associated with seeking care (playing down the need for medical help and the perception of a lack of response from health services) may be the most important individual factors influencing changes in access to health care [37]. Similarly, the status of health professionals in the Spanish context may have led to greater adherence by the population to the recommendations made to stay at home.

Other factors which could respond to our findings are those related to sociodemographic characteristics. There is a reported downturn in healthcare use among the female population [38] and in ethnic minorities and low-income users [39]. However, in Spain, notwithstanding some patients could seek private care to avoid delays, the out-of-pocket healthcare costs could not be a major barrier in Spain because the public healthcare system generally ensures the medical care access without co-payments, as the system is funded through taxes and is independent of the individual’s use of services. Nevertheless, there is a lack of evidence-based information about the barriers to access the services that should be addressed [40].

However, regardless of the initial management of the pandemic, and in line with the main objective of this study, which focused on the perception of Long-COVID patients towards the healthcare system, it is necessary to approach the debilities that have been shown by Long COVID patients in this study. Considering this population as patients with a recognized disease who need a therapeutic approach [41] is a health challenge in the short and medium term that should be addressed by the health systems. In this sense, a comparative analysis of healthcare systems is necessary to understand how they can better manage long-term conditions such as Long COVID [32,42,43].

Long COVID symptoms persist and significantly impact daily life, with patients experiencing severe fatigue, muscle and joint pain, cognitive issues, cardiovascular and respiratory symptoms, dermatological problems, and psychological distress. These findings are consistent with other studies that have documented that a wide range of Long COVID symptoms that affect patients’ physical, neurological, and emotional well-being [44]. In addition, patients felt that the care they received was heavily influenced by the observability of their symptoms. When symptoms were subjective, they perceived a barrier in receiving adequate care. This sentiment has been echoed in studies on chronic illnesses such as fibromyalgia, where patients often face skepticism and underestimation of their symptoms by healthcare providers [45], similar to Long COVID [46].

The variation in self-reported health status among participants highlights the diverse experiences and ongoing health challenges faced by Long COVID patients. However, the fact that almost all participants have not fully recovered from the disease and report a significant decline in their perceived health status is consistent with previous findings [47,48,49] and reveals the significant impact that Long COVID has on patients’ health.

With respect to patients’ perceptions of healthcare systems, the participants had mixed experiences with primary and specialized healthcare. While some were satisfied with their personal interactions with their physicians, many were frustrated with systemic inefficiencies and delays. This frustration was particularly pronounced regarding the impossibility of personal treatment and long waiting times for appointments. The psychological impact of Long COVID and the perceived lack of support from healthcare providers were significant issues contributing to feelings of abandonment and distress. These findings are consistent with previous research indicating the importance of timely and empathetic care in managing chronic illnesses [46,50]. Some patients, as previously reported [48], turned to private healthcare due to the limitations of the public health system in providing timely responses to their needs. This shift to private care has also been observed in other studies, particularly when patients feel neglected or underserved by public healthcare services [46].

On the other hand, the study suggests that nursing may be underutilized in the provision of Long COVID care, as most participants reported minimal interactions with nursing staff, often limited to tasks such as blood draws or vaccine administration. This contrasts with the situation during the pandemic, when, in Spain, nurses’ work in primary healthcare was very important, conferring them more visibility and empowerment [51,52]. This approach would also fully align with the proposal for a multidisciplinary and holistic healthcare response, such as specialized Long COVID units. The participants who accessed these services generally had positive evaluations. However, barriers to access, such as stringent selection criteria and a lack of information among primary care doctors, were significant issues. This reveals the need for better communication and coordination within the healthcare system to ensure that patients receive appropriate care [53].

The participants strongly emphasized the need for shorter waiting times for medical appointments, arguing that delays exacerbate their conditions. This demand is in line with findings from other studies that stress the importance of timely healthcare access [32,54]. The creation of more Long COVID units closer to patients’ health areas was another critical demand. These units should be multidisciplinary and include specialists trained in diagnosis, symptoms, and disease progression. The need for coordinated, comprehensive care has been highlighted in numerous studies on chronic and post-acute COVID-19 conditions, underscoring the importance of specialized, integrated care teams [50,55].

Long COVID patients have long emphasized the need to humanize care, with empathetic healthcare teams capable of listening to and understanding their situations. The providing of comprehensive care, including non-pharmacological treatments such as physiotherapy and psychological support, was deemed essential. Indeed, the effectiveness of programs designed to support mental wellbeing and physical health has been shown to be effective [53]. The importance of empathy and holistic care in managing chronic illnesses has been well documented, highlighting the need for healthcare systems to adopt more patient-centered approaches [48].

## 5. Limitations and Strengths

One limitation of this study is a potential selection bias due to the recruitment of participants through Long COVID patient associations, which may not fully represent the broader patient population, particularly those from lower socio-economic backgrounds. Additionally, conducting interviews virtually may have resulted in a reduced interpersonal connection between the informants and the interviewer. Another limitation is that the study did not track participants’ experiences longitudinally, limiting our ability to capture changes over time, which could be an area for future research. Moreover, the findings may not be fully applicable in other international settings or populations, such as children or other healthcare systems.

Despite these limitations, the study has several strengths. The descriptive phenomenological methodology employed was instrumental in eliciting rich insights by focusing on participants’ lived experiences and perceptions, allowing for a deep exploration of their interactions with the healthcare system and the impact of Long COVID. The thematic approach enabled us to identify key aspects of their healthcare experiences and potential areas for improvement.

While the qualitative nature of the study limits the generalizability of the findings, the methodology employed and the number of interviews conducted ensured data saturation and provided valuable insights. Pilot interviews were conducted prior to the main interviews to develop an appropriate interview guide, enhancing the reliability of the data collection process. Furthermore, although there might be potential bias in the representativeness of the participants, the proportion of men and women reflects the higher prevalence of the disease among females. The in-depth interviews offered valuable insights into patient experiences and needs, which are critical for informing healthcare improvements. Additionally, having a single interviewer who completed all the interviews ensured uniformity in the application of the protocol, which strengthened the consistency of the data collection process.

### Implications for Clinical Practice

These findings underscore the need for healthcare systems to develop more responsive and empathetic approaches to the management of Long COVID. This includes reducing waiting times for appointments, improving diagnostic processes, and creating specialized, multidisciplinary care units. Ensuring that healthcare staff are adequately trained and informed about Long COVID is crucial for delivering effective and compassionate care. Integrating patient perspectives into the design of healthcare services can increase their relevance and effectiveness, ultimately improving patient outcomes and quality of life. These implications align with broader recommendations from studies on Long COVID and chronic disease management, emphasizing the importance of patient-centered care, timely access to services, and comprehensive support systems [10,54].

## 6. Conclusions

This study highlights the significant physical and psychological challenges faced by Long COVID-19 patients, emphasizing the need for improved healthcare models that address both health and socioeconomic needs. The findings underscore the importance of timely access to medical care, comprehensive and empathetic healthcare services, and the establishment of specialized Long COVID units. The study also calls for better training and coordination among healthcare providers, the adoption of patient-centered approaches, and the integration of non-pharmacological treatments. By reflecting on patient experiences, the study advocates for a multidisciplinary and holistic approach to managing Long COVID, aligning with broader recommendations for chronic disease management.

## Figures and Tables

**Table 1:** Semi-structured interview guide.

Main Question	Probing Questions
How was your experience when you got infected with COVID-19? What symptoms did you have?	What happened when you stopped testing positive for COVID-19? How did you feel? How did the symptoms evolve? How are you today? What are your symptoms like? And regarding your mental health, has it been affected? In what way? Is there anything that improves or worsens your symptoms? Did you have any other illnesses before contracting COVID-19?
Through which points of the healthcare system have you passed? (Primary Care, Specialized Care…). Are you satisfied with the care you have received?	Have you visited any specific clinics/units for Long COVID? Are you regularly monitored by healthcare professionals? What explanations have you been given by the healthcare system? What therapies have you tried within the healthcare system? What do they consist of? And outside the healthcare system, have you sought help? Are you taking any specific medication for Long COVID? Which ones? Have they worked for you? Is there any therapy that has benefited you or worked for you? And from nursing care, do you receive any attention?
What would good healthcare look like from your point of view, and why?	What information do you think should be provided to people diagnosed with Long COVID? Would you like the attention in consultations to be different?

**Table 2:** Coding table.

Theme	Categories	Codes
Healthcare	COVID-19 infection	Symptom management Access to testing Progression and follow-up
	Long COVID	Diagnosis
		Healthcare assistance
		Perceived satisfaction
	Demands and improvements	Necessary information

**Table 3:** Sociodemographic data.

Variable	Categories	Frequency (Percentage)
Sex	Female	18 (66.67%)
	Male	9 (33.33%)
Age	30–39	3 (11.11%)
	40–49	9 (33.33%)
	50–59	10 (37.04%)
	60–69	5 (18.52%)
Civil status	Married	16 (59.26%)
	Single	6 (22.22%)
	Separated/Divorced	3 (11.11%)
	Commonlaw Partner	2 (7.41%)
Highest level of education completed	Tertiary Education	15 (55.56%)
	Secondary Education	8 (29.63%)
	Primary Education	2 (7.41%)
	No answer	2 (7.41%)
Current employment status	Working but on leave for more than 3 months	16 (59.26%)
	Working	5 (18.52%)
	Permanently Disabled	3 (11.11%)
	Retired due to age	1 (3.70%)
	Unemployed with subsidy or benefit	1 (3.70%)
COVID-19 diagnosis	PCR Test	18 (66.67%)
	Rapid Antigen Test	6 (22.22%)
	Medical Diagnosis (no test)	2 (7.41%)
	Antibody Test (blood)	1 (3.70%)
Severity of symptoms upon infection	Mild	9 (33.33%)
	Severe but at home	14 (51.85%)
	Hospitalized	4 (14.81%)

## Data Availability

The data are unavailable due to privacy and ethical restrictions. The data will be available upon request, with permission for the purposes of peer review.

## Public Involvement Statement

Participants were involved through semi-interviews, which were conducted and recorded via videoconference using Microsoft Teams Classic. The consent form was previously sent to them for dissemination after they were informed about the objectives and procedures of the study. Similarly, once our results are disseminated through scientific publications, they can be disseminated through social networks and to populations affected by Long COVID.

## Guidelines and Standards Statement

This manuscript was drafted following the COREQ (Consolidated Criteria for Reporting Qualitative Research) for qualitative research.

## Appendix A

**Table A1:** Long COVID persistent symptoms—frequency and intensity.

Symptoms	Not Suffered	Mild	Moderate	Intense
General discomfort	2 (7.41%)	0 (0.00%)	11 (40.74%)	14 (51.85%)
Fatigue	1 (3.70%)	1 (3.70%)	4 (14.81%)	21 (77.78%)
Muscle/joint pain	1 (3.70%)	2 (7.41%)	6 (22.22%)	18 (66.67%)
Cough	4 (14.81%)	10 (37.04%)	7 (25.93%)	6 (22.22%)
Shortness of breath (dyspnea)	4 (14.81%)	2 (7.41%)	11 (40.74%)	10 (37.04%)
Diarrhea	13 (48.15%)	8 (29.63%)	4 (14.81%)	2 (7.41%)
Skin rashes	13 (48.15%)	6 (22.22%)	5 (18.52%)	3 (11.11%)
Hair loss	12 (44.44%)	2 (7.41%)	8 (29.63%)	5 (18.52%)
Headache	4 (14.81%)	1 (3.70%)	7 (25.93%)	15 (55.56%)
Difficulty concentrating/Brain fog	0 (0.00%)	3 (11.11%)	6 (22.22%)	18 (66.67%)
Memory loss	1 (3.70%)	4 (14.81%)	9 (33.33%)	13 (48.15%)
Loss of taste Loss of smell	13 (48.15%)	5 (18.52%)	6 (22.22%)	3 (11.11%)
	14 (51.85%)	3 (11.11%)	7 (25.93%)	3 (11.11%)
Mood alterations (anxiety, depression)	5 (18.52%)	1 (3.70%)	11 (40.74%)	10 (37.04%)
Palpitations	1 (3.70%)	3 (11.11%)	12 (44.44%)	9 (33.33%)
Difficulty swallowing	9 (33.33%)	8 (29.63%)	7 (25.93%)	3 (11.11%)
Conjunctivitis	13 (48.15%)	7 (25.93%)	5 (18.52%)	2 (7.41%)
